# It's time to stop thinking about obesity treatment as an anti-vaxxer

**DOI:** 10.20945/2359-3997000000409

**Published:** 2021-10-29

**Affiliations:** 1 Hospital das Clínicas da Faculdade de Medicina da Universidade de São Paulo São Paulo SP Brasil Liga de Obesidade Infantil do Hospital das Clínicas da Faculdade de Medicina da Universidade de São Paulo (HC-FMUSP). Grupo de Obesidade e Síndrome Metabólica do HC-FMUSP, São Paulo, SP, Brasil

There is no poetry in living with obesity, although it is one of the most prevalent diseases in modern society. Subjects with obesity tend to blame themselves for their condition, along with their family, friends and even health professionals. The “easy and simple” solution in the words of most of them is: “eat less and exercise more!” Based on this old big two-theory, this mindset ignores the complex and multifactorial etiology of obesity (1).

The sooner and more severe the obesity, higher is the reduction in life expectancy and, further on, more years of life with disabilities to face (2). This implies that adolescents with obesity would experience lifelong health impairments and, mainly, psychological suffering. So, when obesity prevention fails, there is enough evidence for treating obesity in these patients.

In this issue of the *Archives of Endocrinology and Metabolism*, Cominato and cols. present a concise, but complete review about obesity treatment in adolescents. Lifestyle interventions, medications and bariatric surgery are discussed with randomized clinical trials and meta-analysis to support it are presented (3). These types of trials offer an evidence level A, but when obesity treatment is the issue, this is not very well accepted for many health care professionals or researchers.

It is well established that obesity is related to many comorbidities, worsening quality of life, and decreasing life expectancy. In adults, weight loss medications improve comorbidities and quality of life, as does bariatric surgery, which also reduces mortality. Unfortunately, these treatments are not only unavailable for most subjects with obesity seeking for help, but they are also demonized, worsening physical and mental disorders of these patients. If obesity treatment for adults is a challenge, for adolescents it is even more difficult, because we need scientific evidence to support the best care to this patients.

The authors update us on drugs used to treat obesity associated to lifestyle changes. Some medications are well studied in adolescents with obesity, but not all are already approved to be used in this group.

Recently, liraglutide was approved in Brazil and the USA for obesity treatment in adolescents. It is noteworthy that this is a high-cost medication, with subcutaneous daily use, which can be a barrier.

The total number of adolescents with obesity studied in the liraglutide trial is lower than the amount evaluated in sibutramine and orlistat trials, and the efficacy does not seem to be much higher. It is also worth mentioning that liraglutide has a better cardiovascular and psychiatric safety profile than sibutramine, and that orlistat does not seem to be as effective (3).

The Endocrine Society guidelines recommend weight loss medications when lifestyle interventions fail in the treatment of adolescents with a BMI z-score higher than +2, recommending against the use of medications for simply overweight adolescents. It is also important to remember that if the patient doesn't achieve a weight reduction >4% in BMI or BMI z-score after taking the antiobesity medication in full dosage for 12 weeks it should be stopped (4).

Bariatric surgery leads to significant and sustained weight loss in adults and adolescents. The best time and criteria to refer adolescents for surgical treatment is not well defined. But bariatric surgery changes lives and is a treatment option for adolescents with more severe obesity. The cohort of Teen-LABS Consortium, in which 161 patients aged 13 to 17 years with a mean BMI of 50 kg/m^2^, showed that the chance of resolution of comorbidities is higher in teens than in adults. Despite the rate of abdominal reoperations having been significantly higher among adolescents than adults, the mortality rate was similar. As pointed in Cominato's paper (3), obesity in adulthood is related to obesity in childhood, and probably this is much truer in patients with extremely severe obesity. Note that the treatment will not cure, neither solve these cases of obesity, but it will control the disease: after five years, in adolescents of Teen-LABS, the mean BMI was 37 kg/m^2^, which is praiseworthy (5).

Finally, lifestyle modifications are, of course, always the first and endless approach, even when drugs and/or surgery are used, because the health care of people with obesity is not only about losing weight, but about improving global health. Obesity prevention strategies are ineffective worldwide, as the numbers of overweight and obesity increase, especially in this pandemic COVID-time (6). Lifestyle modifications usually decrease BMI z-score in about 0.3, which is a mild effect (7). For other diseases, like type 1 diabetes, for example, food choice and control of ingestion of carbohydrates are recommended with established criteria. Dietary recommendations in obesity management used to be generalist, and maybe this absence of more specific guidance in a world with plenty of hyperpalatable foods, sold with insistent marketing, is almost impossible to follow and impractical to maintain a healthier diet in the long term.

With so many studies already published showing efficacy and safety of weight loss medications and bariatric surgery, added to lifestyle modifications, to stigmatize these obesity treatments sounds like anti-vaccine movement and its fake news: they choose what they want to believe and share it, despite science. It is time to accept the scientific evidence, which will improve the respect and the care for people with obesity.
